# Assessing anaesthesiology and intensive care specialty physicians: An Italian language multisource feedback system

**DOI:** 10.1371/journal.pone.0250404

**Published:** 2021-04-23

**Authors:** Luca Carenzo, Tiziana Cena, Fabio Carfagna, Valentina Rondi, Pier Luigi Ingrassia, Maurizio Cecconi, Claudio Violato, Francesco Della Corte, Rosanna Vaschetto

**Affiliations:** 1 Department of Anesthesia and Intensive Care Medicine, Humanitas Clinical and Research Center—IRCCS, Rozzano (MI), Italy; 2 Department of Anaesthesia and Intensive Care Medicine, Azienda Ospedaliero-Universitaria “Maggiore della Carità”, Novara, Italy; 3 Department of Biomedical Sciences, Humanitas University, Pieve Emanuele–Milan, Italy; 4 Dipartimento di Medicina Traslazionale, Università del Piemonte Orientale, Novara, Italy; 5 Centro di Simulazione, Centro Professionale Sociosanitario, Lugano, Switzerland; 6 Centro Interdipartimentale di Didattica Innovativa e di Simulazione in Medicina e Professioni Sanitarie, SIMNOVA, Università del Piemonte Orientale, Novara, Italy; 7 Departments of Medicine and Medical Education, University of Minnesota Medical School, Minneapolis, MN, United States of America; University of Palermo, ITALY

## Abstract

**Background:**

Physician professionalism, including anaesthesiologists and intensive care doctors, should be continuously assessed during training and subsequent clinical practice. Multi-source feedback (MSF) is an assessment system in which healthcare professionals are assessed on several constructs (e.g., communication, professionalism, etc.) by multiple people (medical colleagues, coworkers, patients, self) in their sphere of influence. MSF has gained widespread acceptance for both formative and summative assessment of professionalism for reflecting on how to improve clinical practice.

**Methods:**

Instrument development and psychometric analysis (feasibility, reliability, construct validity via exploratory factor analysis) for MSF questionnaires in a postgraduate specialty training in Anaesthesiology and intensive care in Italy. Sixty-four residents at the Università del Piemonte Orientale (Italy) Anesthesiology Residency Program. Main outcomes assessed were: development and psychometric testing of 4 questionnaires: self, medical colleague, coworker and patient assessment.

**Results:**

Overall 605 medical colleague questionnaires (mean of 9.3 ±1.9) and 543 coworker surveys (mean 8.4 ±1.4) were collected providing high mean ratings for all items (> 4.0 /5.0). The self-assessment item mean score ranged from 3.1 to 4.3. Patient questionnaires (n = 308) were returned from 31 residents (40%; mean 9.9 ± 6.2). Three items had high percentages of “unable to assess” (> 15%) in coworker questionnaires. Factor analyses resulted in a two-factor solution: clinical management with leadership and accountability accounting for at least 75% of the total variance for the medical colleague and coworker’s survey with high internal consistency reliability (Cronbach’s α > 0.9). Patient’s questionnaires had a low return rate, a limited exploratory analysis was performed.

**Conclusions:**

We provide a feasible and reliable Italian language MSF instrument with evidence of construct validity for the self, coworkers and medical colleague. Patient feedback was difficult to collect in our setting.

## Background

Evaluation and upkeep of doctor’s professional fitness is relevant to colleges, licensing authorities, patients, co-workers and the public. The idea of medical competence, and its assessment, has significantly shifted from the simple ability of performing medical interventions into the broader concept of overall physician performance including interpersonal skills and professionalism. Multisource feedback (MSF), also known as “360-degree assessment” is a tool for assessing professional fitness, manners and attitudes in the workplace [1].

MSF has been used in many countries such as Canada, the United Kingdom, the United States, Netherlands and others to assess aspects of physician professionalism, communications, medical expertise, collegiality, health advocacy and systems-based practice [2]. In a recent systematic review of physician performance assessment with MSF, the majority of the studies provided evidence of the construct validity of the MSF instruments as well as evidence of reliability and generalizability [2].

In Italy, post-graduate medical education takes place in healthcare facilities of the National Health System (NHS) but is provided by universities through residency programs, under the supervision of the University and Research Ministry (MIUR). After a revision for all post-graduate core curricula, MIUR and Health Ministry have recently established a new accreditation system for the residency programs, which imposes the adoption of a quality management system to register the educational and training activities dedicated to the residents and to assess and certify knowledge, skills, and attitudes achieved by every single resident at the end of the program [3]. The training in Anesthesiology and Intensive Care Medicine are brought together in a combined program which follows either the national rules and regulations and the comprehensive guidelines on the minimum European Training Requirement (ETR) published by the European Board of Anaesthesiology (EBA), and the European Board of Intensive Care Medicine, both members of the European Union Medical Specialties (UEMS) [4–6]. Both of them also recommend the need for specific training and assessment regarding professionalism in the field of Anesthesiology and Intensive Care Medicine.

There are many definitions of professionalism. The Association of American Medical Colleges states that physicians must be altruistic, knowledgeable, skillful and dutiful—all attributes of professionalism [7]. Teaching and assessing professionalism is today more important than ever both because it is prescribed by regulation, and because patients, co-workers and the public expect physicians to be professional. Medical societies also expect professionalism to be taught and assessed. Moreover, professionalism is associated with improved medical outcomes and literature suggests that unprofessional behaviors can be associated with medical errors and adverse outcomes [8, 9].

Competencies such as professionalism, should be acquired within defined time-frames and learning objectives should be continuously assessed during training. The assessment of professionalism in medicine is important but challenging. Historically, assessments have been implicit, unstandardized, and based on holistic or subjective judgments (the apprenticeship model) [10]. Although many forms of assessment can be used to show a doctor’s knowledge or competence, there is not much evidence that competence is systematically related to performance in clinical practice [11].

Recent reforms in postgraduate medical education [12] have brought new systems for the assessment of competence and performance. Workplace-based assessment is one of these systems. Workplace-based assessment refers to “the assessment of day-to-day practices undertaken in the working environment” [13]—or, more simply, is an “assessment of what doctors actually do in practice.” One major advantage of workplace-based assessment is its ability to evaluate performance in context [14]. Multi-source feedback (MSF) systems contain tools commonly used in workplace-based assessment, particularly suitable for assessing professionalism, and can be used for both developmental and appraisal purposes as well as to facilitate change for both the professional and the organization.

In MSF physicians complete a self-assessment questionnaire and receive feedback from a number of medical colleagues (both supervisors and peers), non-physician coworkers (e.g., nurses, physiotherapists, pharmacists, etc.), as well as their own patients on a number of items relevant to the explored topic. Subsequently, the physician receives aggregate anonymous feedback about performance [15, 16]. Different respondents focus on characteristics of the physician that they can assess (e.g., patients are not expected to assess a physician’s clinical expertise) and together provide a more comprehensive evaluation than what could be derived by any one source alone [17]. Previous studies suggest that patient’s feedback is important, as it allows to assess doctors’ communication skills, which involve a combination of conscious and unconscious processes and responses [18], and their ability to inspire trust and to make the patient feel involved in the decision- making about their treatment [19, 20].

MSF originated from work in business [21]. Their use in medical education has been extensively reviewed by Donnon et al. [2] and they have already been successfully applied in a series of medical specialties. Completion of several MSF assessments is now a requirement for physicians in the UK [22] and in general they have widespread acceptance in the United States, Canada, United Kingdom and the Netherlands for evaluation of professionals, where they are seen as a catalyst for the practitioner to reflect on where change may be required [23–29]. Although Italy is recently witnessing a strong movement toward a competency-based education at the postgraduate level, highlighting the need for reliable assessment methods and instruments to verify the achievement of certain level of competencies, only a few initiatives incorporate MSF in the formal assessment strategy [30]. To our knowledge, no such assessment system is available in the Italian language for the practice of anesthesiology.

The main aim of the present study then was to develop a new Italian language multi-source feedback system for the practice of anesthesiology and intensive care medicine. Accordingly, it was necessary to perform psychometric analyses (feasibility, reliability, construct validity via exploratory factor analysis) of MSF instruments developed for this purpose.

## Methods

### Participants

All active residents (n = 70) in the program at the Università del Piemonte Orientale, Novara, Italy Anesthesiology Residency Program during the academic year 2017/2018 were invited to participate in the study which lasted between March 2019 and September 2019. A total of 64 (91.4%) participated in the study.

### Procedures

#### Questionnaire development

A working group composed of four content experts (faculty from the school of anesthesiology) and three methodologists (a medical educational expert and two statisticians) was tasked to review previously published instruments and other tools currently used by other anesthesiology societies in the world. Taking inspiration from the instruments designed by Lockyer et al. [23], which had been found to be reliable and valid, the group developed a set of instruments for Italian anesthesiologist residents with the aim of adapting the items from this instrument to the local anesthesiology training programs based on the characteristics’ framework used by Yamamoto et al. [31]. The final agreement of the experts on the selected items provided the initial content validity for the tool following Wood et al. [32] on MSF development and implementation.

Based on the experts’ responses and comments, questions were revised and modified and finalized into the four typical components of multi-source feedback: medical colleague, non-medical coworker, self-assessment and patient questionnaires. The instrument for medical colleague and coworker consisted of 17 items, with a 5-point rating scale (1 = poor to 5 = excellent). The self-assessment questionnaire was identical to the medical colleague and coworker questionnaire, but items were written in the first person. The patient questionnaire consisted of 12 items on a five-point rating scale ranging from 1 = strongly disagree to 5 = strongly agree. The possibility of answering “unable to assess” for each question was available throughout. Questionnaires are provided as online supplemental data files.

#### Questionnaire testing

Medical colleague, non-medical coworker and self-questionnaires were loaded onto an online survey platform. Each study participant received the invite for the self-questionnaire in his academic inbox. After answering the self-assessment, links for the medical colleague and non-medical coworker questionnaires were sent out individually to the residents. Participants were requested to complete a minimum of 8 medical colleague (including both supervisors and peers) questionnaires and 8 non-medical coworker questionnaires. This was based on a previous review that suggested that a minimum of 8 medical colleagues and 8 coworkers’ responses are needed to achieve adequate reliability and generalizability. The same data suggests optimal reliability from the patients’ questionnaire with 25 forms per participant [2]. The number of patients’ questionnaires asked to be collected was reviewed and was reduced from 25 to 12 to improve the feasibility of maximizing the number of questionnaires per resident, following feedback that the overall participants perception was that the patient’s questionnaire would be difficult to obtain. Questionnaires did not ask for personal identifiers of the evaluators. Previous works had established that raters chosen by people being assessed do not provide significantly different evaluations than those selected by a third party [33]. Furthermore, studies examining how well the assessor and assessed physician know one another show that “familiarity” contributes very little to the variance in ratings [24, 34].

#### Feedback

Feedback was generated for each trainee to provide evidence for the in-training assessment process and to support personal development planning. It was explained that forms would be anonymously merged before being returned to individual residents, and that results would not be included, at this stage, in residents’ portfolios.

#### Data analyses

Descriptive statistics (mean, standard deviation or median and quartiles) were used to summarize scores of the items and scales. Response rates for each question for each instrument were measured to determine the feasibility of different respondent groups.

For each item, the mean and standard deviation was computed along with the percentage of “unable to assess”. Different sources suggest that an item with a rate of unable to answer response rate above 15% or 20% might suggest the need for reassessment or cancellation of the question [23]. In this study we used 15% as the cut-off value. Score profiles for each of the items (i.e., mean and standard deviation) on the surveys are presented for every questionnaire.

Exploratory factor analysis was employed to identify factors and number of factors for each instrument: A Pearson’s correlation matrix was decomposed into principal axis retaining factors with eigenvalues ≥1. Promax rotation retaining loadings of absolute value ≥0.32 was used to identify a simple component structure [35].

Internal consistency reliability, Cronbach’s 〈 coefficient, for each of the factors for each of the instruments was calculated. This provided an indication of whether factors have overall cohesiveness. This analysis was followed by a generalizability analysis to determine the generalizability coefficient (Ep^2^) to ensure there were sufficient numbers of items and raters to provide stable data for individual anesthesiologists on every instrument. An Ep^2^ ≥0.70 indicates adequate generalizability.

Statistical significance was set at p<0.05. The statistical analysis was performed using R version 3.6.1 [36].

### Ethics

Ethical approval for this study (prot. 152/CE, studio 30/19) was provided by the Ethical Committee Comitato Etico Interaziendale A.O.U. “Maggiore della Carità”, ASL BI, ASL NO, ASL VCO, Novara, Italy (Chairperson Prof Pier Davide Guenzi) on 04 March 2019. Each participant provided written informed consent. Data was anonymized and identified by means of random identifiers, it did not contain any participant characteristics except for post-graduate year in the self-assessment. In addition, the study was conducted in accordance with the guidelines stated in the Standards for Quality Improvement Reporting Excellence (SQUIRE) [37].

## Results

Sixty-four residents provided a self-assessment questionnaire. For all of them (100%) the minimum required amount of medical and non-medical feedback questionnaires was returned resulting in 605 medical colleague questionnaires (mean of 9.3 ±1.9 per participant) and 543 coworker surveys (mean 8.4 ±1.4 per participant) were collected. Only 308 (40% of expected) patient questionnaires were returned from 31 residents (mean 9.9±6.2).

The majority of items on the questionnaires could be answered by respondents. Regarding the self-questionnaire, the “unable to assess” category ranged from 0.00% to 3.08%. The question with the highest percentage of “unable to assess” was "ability to multitask and work effectively in a complex environment". The “unable to assess” category for questions from the medical colleague and the coworker questionnaires ranged from 0.17% to 22.06%. Three items in the non-medical coworker questionnaires resulted in >15% of unable to assess answers. The three items were: ability in delivering effective handovers (15.14%), ability in providing honest and constructive feedback (15.33%) and ability to take a leadership role when circumstances required (22.06%).

Both the medical and non-medical colleague data indicated that the mean score for all items was greater than 4.0 out of 5. The self-assessment scores the participants provided were lower on all items when compared to the same questions answered by medical colleagues and non-medical co-workers, with means ranging from 3.1 for the item on being able to step up as a leader when deemed necessary to 4.30 for respecting the rights and privacy of patients.

### Factor analysis

Two factors emerged from the factor analysis for the three instruments. The items aligned into two broad domains (factors): clinical management with leadership and accountability. In the self-questionnaire the two factors explain 42.2% of the total variance and the two dimensions are moderately correlated (0.46). The Cronbach’s α for the first factor is 0.87, while 0.81 for the second factor. In the medical colleague and non-physician coworker’s questionnaire the 2-factor solution explained 75% of the variance. The two dimensions have a high correlation (0.81), suggesting that a high score in one dimension could be related to a high score in the second dimension.

The Cronbach α coefficients for the factors for the medical colleague and non-medical colleague instruments were high, all above 0.90. Generalizability coefficients (Ep^2^) were respectively 0.98 and 0.94 and 0.97 and 0.92 for the first and second factor of the medical colleague and non-medical co-workers questionnaire.

**Fig 1:**
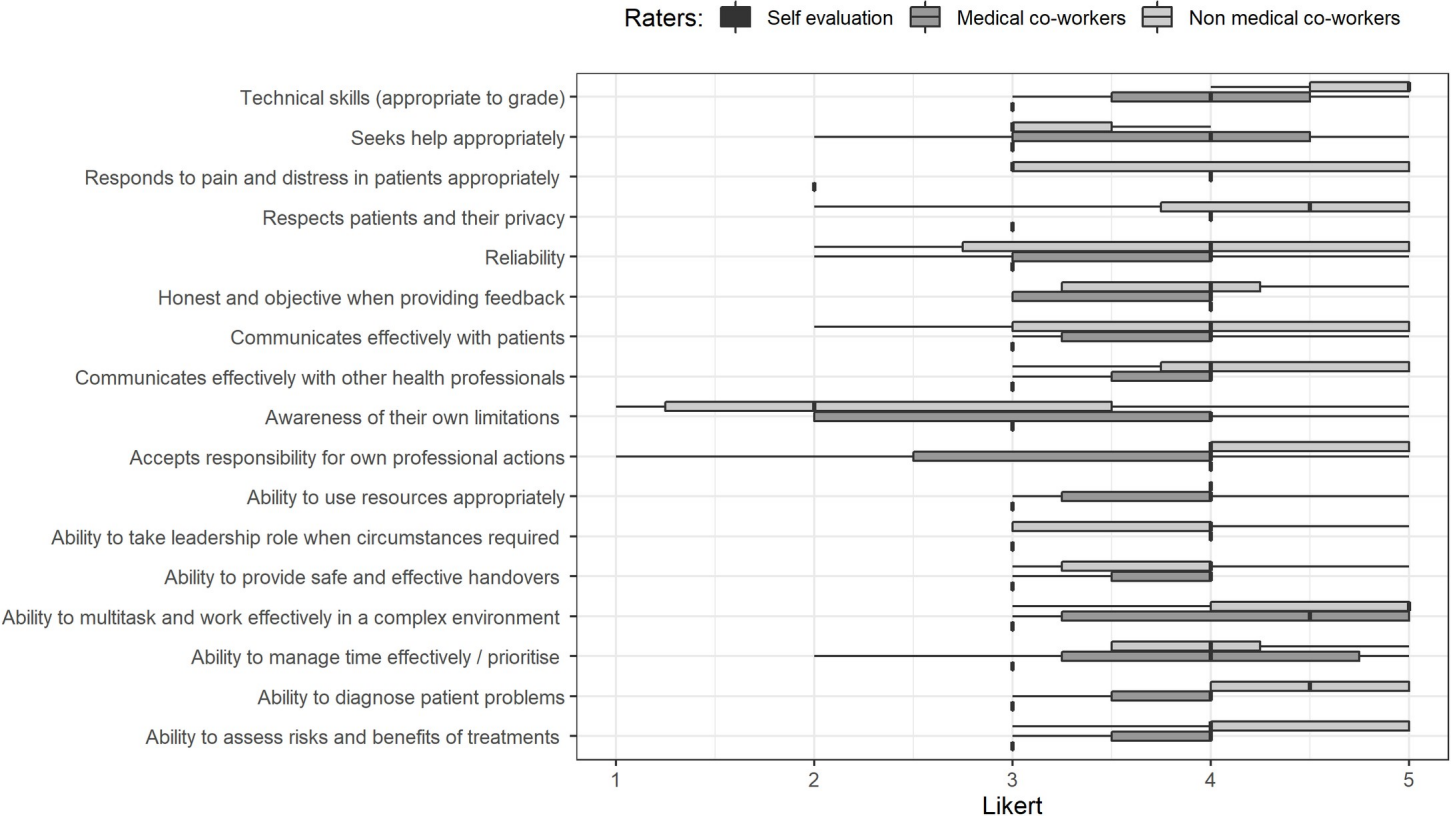
An example of the graphical feedback provided to each resident.

Details about questions, response rate, range and unable to assess rate, factor analysis and Cronbach’s α for the, medical colleague, coworker, self and patient questionnaires are presented in Tables 1, 2, 3 and 4 respectively. Although the primary aim of this study was the development and psychometric analyses of an Italian language MSF instrument, it was a good opportunity to provide individual feedback to participating residents. Fig 1 shows an example of the graphical feedback provided to each resident, with the plot of their self-assessment compared to their colleagues and co-worker’s assessment and the relative range.

**Table 1:** Details about questions, response rate and unable to assess rate, factor analysis and Cronbach α scores for medical colleagues.

	ITEMS	N	MEAN	S.D	% U/A	FACTOR 1 Management and Leadership	FACTOR 2 Accountability
A	Ability to diagnose patient problems	590	4.3	0.85	0.84%	0.84	
B	Technical skills (appropriate to grade)	585	4.3	0.85	1.68%	0.96	
C	Ability to assess risks and benefits of treatments	587	4.3	0.82	1.34%	0.80	
D	Responds to pain and distress in patients appropriately	581	4.4	0.77	2.35%	0.47	0.40
E	Communicates effectively with patients	574	4.4	0.76	3.53%		0.50
F	Communicates effectively with other health professionals	592	4.3	0.92	0.50%		0.69
G	Ability to provide safe and effective handovers	579	4.4	0.89	2.69%	0.63	
H	Honest and objective when providing feedback	582	4.3	0.92	2.18%	0.52	0.40
I	Respects patients and their privacy	593	4.7	0.59	0.34%		0.55
J	Seeks help appropriately	580	4.4	0.82	2.52%		0.90
K	Awareness of their own limitations	570	4.3	0.95	4.20%		1.00
L	Accepts responsibility for own professional actions	582	4.4	0.85	2.18%		0.80
M	Reliability	594	4.4	0.93	0.17%	0.49	0.48
N	Ability to multitask and work effectively in a complex environment	589	4.2	0.98	1.01%	0.78	
O	Ability to manage time effectively / prioritise	580	4.2	0.92	2.52%	0.90	
P	Ability to use resources appropriately	576	4.3	0.83	3.19%	0.85	
Q	Ability to take leadership role when circumstances required	557	4.1	1.06	6.39%	0.90	
	Cronbach α					0.97	0.92

n = number of responses, S.D = Standard Deviation, U/A = Unable to assess.

**Table 2:** Details about questions, response rate and unable to assess rate, factor analysis and Cronbach α scores for coworkers.

	ITEMS	N	MEAN	S.D	% U/A	FACTOR 1 Management and Leadership	FACTOR 2 Accountability
A	Ability to diagnose patient problems	463	4.4	0.82	13.46%	0.82	
B	Technical skills (appropriate to grade)	499	4.4	0.79	6.73%	1.07	
C	Ability to assess risks and benefits of treatments	468	4.3	0.84	12.52%	0.88	
D	Responds to pain and distress in patients appropriately	478	4.4	0.79	10.65%	0.55	0.34
E	Communicates effectively with patients	518	4.5	0.83	3.18%	0.52	0.35
F	Communicates effectively with other health professionals	516	4.5	0.86	3.55%	0.44	0.45
G	Ability to provide safe and effective handovers	454	4.4	0.79	15.14%	0.60	
H	Honest and objective when providing feedback	453	4.4	0.90	15.33%	0.62	
I	Respects patients and their privacy	514	4.6	0.71	3.93%		0.51
J	Seeks help appropriately	466	4.4	0.90	12.90%		0.96
K	Awareness of their own limitations	459	4.3	0.95	14.21%		0.99
L	Accepts responsibility for own professional actions	458	4.5	0.82	14.39%		0.74
M	Reliability	513	4.4	0.91	4.11%		0.62
N	Ability to multitask and work effectively in a complex environment	482	4.3	0.88	9.91%	0.54	
O	Ability to manage time effectively / prioritize	465	4.2	0.90	13.08%	0.66	
P	Ability to use resources appropriately	470	4.3	0.87	12.15%	0.68	
Q	Ability to take leadership role when circumstances required	417	4.0	1.06	22.06%	0.77	
	Cronbach α					0,98	0,95

n = number of responses, S.D = Standard Deviation, U/A = Unable to assess.

**Table 3:** Details about questions, response rate, and unable to assess rate, factor analysis and Cronbach α scores for self-questionnaire.

	ITEMS	N	MEAN	S.D	% U/A	FACTOR 1 Management and Leadership	FACTOR 2 Accountability
A	Ability to diagnose patient problems	64	3.4	0.61	0.00%	0.75	
B	Technical skills (appropriate to grade)	63	3.5	0.71	1.54%	0.41	
C	Ability to assess risks and benefits of treatments	64	3.5	0.66	0.00%	0.54	
D	Responds to pain and distress in patients appropriately	64	3.3	0.66	0.00%		0.38
E	Communicates effectively with patients	64	3.9	0.71	0.00%	0.60	
F	Communicates effectively with other health professionals	64	3.7	0.67	0.00%	0.48	
G	Ability to provide safe and effective handovers	64	3.5	0.73	0.00%	0.65	
H	Honest and objective when providing feedback	64	3.5	0.77	0.00%	0.71	
I	Respects patients and their privacy	64	4.5	0.81	0.00%		0.76
J	Seeks help appropriately	64	4.3	0.83	0.00%		0.80
K	Awareness of their own limitations	64	4.4	0.76	0.00%		1.01
L	Accepts responsibility for own professional actions	64	4.3	0.78	0.00%		0.46
M	Reliability	64	4.1	0.65	0.00%	0.41	0.44
N	Ability to multitask and work effectively in a complex environment	62	3.4	0.59	3.08%	0.54	
O	Ability to manage time effectively / prioritize	64	3.4	0.67	0.00%	0.69	
P	Ability to use resources appropriately	63	3.4	0.68	1.54%	0.57	
Q	Ability to take leadership role when circumstances required	63	3.1	0.82	1.54%	0.71	
	Cronbach α					0.87	0.81

n = number of responses, S.D = Standard Deviation, U/A = Unable to assess.

**Table 4:** Details about questions unable to assess rate, mean and standard deviation for patient questionnaires.

	ITEMS	N	MEAN	S.D.	% U/A
A	Introducing themselves to you	307	4.8	0.50	0,32%
B	Being polite	306	5.0	0.24	0,64%
C	Putting you at ease	307	4.9	0.28	0,32%
D	Being considerate and scrupulous	303	4.9	0.43	1,6%
E	Explaining clearly	308	4.9	0.32	0%
F	Involving you in the decisions about your anaesthetic/treatment	306	4.9	0.43	0,6%
G	Answering your questions	302	4.9	0.34	1,9%
H	The Anesthetist seems approachable	301	4.9	0.28	2,2%
I	I have confidence in the ability of this anaesthetist to provide safe care	296	4.9	0.32	3,8%
J	I was satisfied with the anaesthetist and would be happy to see him/her again	285	4.9	0.34	7,4%
L	The anaesthetist treated me with dignity and respect	308	5.0	0.24	0%
M	I was given enough privacy by the Anaesthetist	303	5.0	0.23	1,6%

n = number of responses, S.D Standard Deviation, U/A Unable to assess.

## Discussion

In this study we present the development and the performance of an exploratory psychometric analysis of an Italian language questionnaire-based assessment, which combines feedback from medical colleagues, coworkers and patients for assessing anesthesiologists. The questionnaire proved to have a good response rate in both the self-assessment and the colleague’s forms, with most residents taking part in the study, and with all of the study participants meeting the expected questionnaire return rate except for the patients’ feedback questionnaire. The response rates obtained in this study are consistent with response rates for other groups of physicians who have been studied [23, 24].

### Self, medical colleague and non-medical coworker assessment

The majority of items could be responded to by the anesthesiologist’s assessors, with some items which proved difficult for respondents to assess. We found a difference in the ability to answer questions between non-medical coworkers and medical colleagues. Consistent with other MSF studies [23], it may be that some co-workers might not have the occasion to observe residents during a specific behaviors or action. Alternatively, non-medical assessors in this setting might not be appropriately trained to identify specific non-clinical characteristics of doctors, or even might not consider as part of their role as co-worker the assessment of medical colleagues on their professionalism and humanistic characteristics.

Assessing the ability to accept leadership when needed was the most difficult item to assess for nonmedical coworkers. Leadership is a well-recognized part of anesthesiology practice [38]; however, in Italy, often trainees are not exposed to formal leadership training during their anesthesia residency, nor are other allied healthcare professionals during their primary training [39]. A recent review on clinical leadership could not find any study from Italy [40]. In addition, while a general consensus emerges about the ends of soft skills in clinical leadership, in Italy leadership in medicine generally seems still to be equated to technical skills [41]. When implementing a feedback system, it is important to assess the capability of the assessor group to evaluate different aspects of humanistic and professional skills, especially the non-clinical ones. Previous MSF studies have shown that non-physicians can reliably assess aspects of humanistic and psychosocial care [42]. It is possible, however, that this was not the case for some of the assessors in the present study. Given the importance of the topic, the question should be retained to determine if interprofessional leadership training results in increased response rates in the future.

Residents consistently rated themselves lower than medical colleagues and non-medical co-workers did on most items. Patients, on the contrary, were the group with the highest rating of participants. Both these findings are consistent with previous findings from MSF instruments with many medical specialties [18, 43].

Factor analyses of the medical-colleague and co-worker questionnaires resulted in a two-factor model, with a very high proportion of variance being represented by the two factors. The first factor is identified by items related to clinical management with leadership, while the second factor is identified by items related to accountability. The two factors have a high correlation (0.81), suggesting that a high score in one is related to a high score on the second factor. These two domains represent the overall construct of professionalism (which includes management, leadership, accountability, etc.) for which the questionnaire was designed. The two-factor model with a moderate correlation is confirmed for the self-assessment questionnaire. This allowed us to prepare and provide feedback reports to participants using the two-factor model. Grouping items into factors helps in the feedback process as the factors can also be used to guide physicians to reflect and improve on global areas (i.e. accountability) rather than on a single item. Three items in the medical colleague, three items in the non-medical coworker and one item in the self-questionnaire presented cross-loading (items significantly loading on both factors). There is no unique accepted strategy to deal with these items. We retained them in the model as we are interested not only in the empirical but also the conceptual support of the instrument. For example we feel that exploring whether a doctor receiving feedback communicates effectively with other healthcare professionals is conceptually relevant; and others willing to use this instrument should be aware that this item has been explored, although in its present form it might not effectively inform any of the two factors. One might choose to delete these items or to rephrase and repeat a new exploratory factor analysis. Overall, the reliability of the colleagues (medical and non-medical) feedback was highly consistent with other MSF research. Moreover, large generalizability coefficients (≥0.80) with 8-assessors is in concordance with previously published instruments involving six to eight (or more) assessors. Reliability of self-assessment was high but slightly less than the colleague’s questionnaire.

### Patient questionnaire

Most multi-source feedback instruments include a component of patient feedback to the assessment. Obtaining patient feedback is perceived to be challenging in the field of anesthesiology, due to the often-brief contacts with patients that anesthesiologists might have, or the difficult or distressing situation patients might often be in when meeting the clinician. Challenges in collecting patients’ feedback are reported in specialty specific literature which however still encourages it and recommends it, due to the relevance of patient’s feedback in improving the anesthesiologists’ knowledge of his/her patient perception [23].

In the present study we could not reach the expected return rate of the patient’s questionnaire from study participants. This was most likely due to both the fact that participants found it time consuming to collect patient feedback, as well as to the higher number of patient questionnaires required.

Interestingly, there was a clear difference in patient’s feedback collection styles, those participants who provided feedback tended to complete the collection of their questionnaire, versus those who did not, or who did not provide any questionnaire at all. We think this might be due not only from the increased workload of collecting the feedback but as well as the motivation for doing so. Those who reached out to families likely collected all the forms suggesting that the limiting factor was participants not reaching out to families rather than family declining participation in completing the feedback form.

It is worth mentioning that this was a study with volunteer participants using their own time to collect feedback forms. In other systems completing a certain number of MSF is a requirement for training or revalidation and trainees might feel more motivated (or obliged) to collect feedback. Another aspect to keep in mind is the great heterogeneity of the services attended by anesthesiologists: participants could collect feedback in both the outpatient and inpatient settings. Previous studies found significant differences in response rates between the two settings, with much higher response rates in the outpatients, due to family accessibility, logistics and predictability of services [44]. In our study we did not explore if those trainees rotating in an inpatient facility (intensive care for instance) had more difficulties in collecting feedback compared to those rotating in the outpatient pre-assessment clinic or pain clinic. We did not formally explore or collect reasons for participants’ lack of completion of the patient questionnaire, but we think assessing the acceptability and feasibility of gathering patients’ feedback would be a reasonable subject to explore in a dedicated future study, aiming at describing individual or system limitations to obtaining this feedback.

Finally, it is worth mentioning that multi-source feedback is just a tool, its effect on personal development depends on its implementation into a larger framework of post-graduate medical education. Success will depend on the overall organization attitude towards positive change. Only a positive and supportive climate from the organization will support a change of behavior in the assessed professional [32].

It is important as well that feedback collected by means of MSF is given to the assessed doctor in a facilitated fashion, either by an appraiser, mentor, facilitator, or coach. This, as well as the quality of this facilitation, has been suggested to have a role in the physicians’ acceptance and subsequent behavioral change by a review of effectiveness of multi-source feedback [45].

### Limitations and strengths

Limitations of the present study include (1) the relatively small sample size of residents (n = 64) who participated and received feedback; (2) low response rate (40%) of the patient questionnaire; and (3) only one training site of residents was employed. A further limitation is the culturally-dependent assumptions on which some of the constructs are based, for instance the “familiarity" contributing very little to the variance of the ratings reported in cited studies should be experimentally confirmed as true also in Italy.

Strengths include that a full set of MSF instruments (self, peer, coworker and patient) were developed and psychometrically analyzed to assess professionalism and other aspects of clinical practice of anaesthesiology and critical care medicine. Future research should focus on studying the correlations (i.e., criterion-related validity) with other kinds of objective assessments (e.g., performance on objective structured clinical exams–OSCEs), global rating scores, clinical experience, or other proficiency exams [46]. The present study should be extended and replicated in other sites of resident training and physician practice.

## Conclusions

A full set of MSF instruments (self, peers, coworkers, patient) of this first Italian version for anesthesiologists is feasible, reliable with evidence of construct validity. Patient feedback was difficult to obtain. Nonetheless, due to the relevance of patient feedback on individual clinical practice, the patient instrument should be employed as much as possible.

The implementation of an MSF system for Italian anesthesiologists as for other anesthesiologists [20, 23], can provide constructive feedback about the relevant domains of clinical practice, management and professionalism directly from those who interact with the clinician on a daily basis and have direct observation of these behaviors. Further study is warranted to assess the external validity of these instruments in evaluating anesthesiologists’ professionalism and other aspects of clinical practice. It is also our hope that it will prompt a cultural change in professionalism and the culture of a competency-oriented 360° assessment. While best approaches to teaching and evaluating professionalism are still debated, assessment and feedback about professionalism is necessary to change practice and behavior.

## Supporting information

S1 File Self, medical and non-medical coworker questionnaire. (DOCX)

S2 File Patient questionnaire. (DOCX)
