# Supplementary material for: Assessing anaesthesiology and intensive care specialty physicians: An Italian language multisource feedback system
Source: PLoS One. 2021 Apr 23;16(4):e0250404. doi: 10.1371/journal.pone.0250404 (PMC8064525; doi:10.1371/journal.pone.0250404)
Supplement: S1 File — (DOCX) [file pone.0250404.s001.docx]

**Assessing Anaesthesiology and Intensive Care Specialty Physicians: An Italian Language Multisource Feedback System**

Luca Carenzo^1^, Tiziana Cena^2^, Fabio Carfagna^3^, Valentina Rondi^4^, Pier Luigi Ingrassia^5,6^, Maurizio Cecconi^1,3^, Claudio Violato^7^, Francesco Della Corte^2,4^, Rosanna Vaschetto^2,4^,

1. Department of Anesthesia and Intensive Care Medicine, Humanitas Clinical and Research Center - IRCCS, Via Manzoni 56, 20089 Rozzano (MI), Italy
2. Department of Anaesthesia and Intensive Care Medicine, Azienda Ospedaliero-Universitaria “Maggiore della Carità”, Via Mazzini 18, 28100 Novara, Italy
3. Humanitas University, Department of Biomedical Sciences, Via Rita Levi Montalcini 4, 20090 Pieve Emanuele – Milan, Italy.
4. Dipartimento di Medicina Traslazionale, Università del Piemonte Orientale, Via Solaroli 17, 28100 Novara, Italy
5. Centro di Simulazione, Centro Professionale Sociosanitario, Via Ronchetto 14, 6900, Lugano, Switzerland
6. Centro Interdipartimentale di Didattica Innovativa e di Simulazione in Medicina e Professioni Sanitarie, SIMNOVA, Università del Piemonte Orientale, Via Lanino 1, 28100 Novara, Italy.
7. Departments of Medicine and Medical Education, University of Minnesota Medical School, 420 Delaware St. SE, Minneapolis, MN, USA, 55455

Corresponding author:

Luca Carenzo,

Department of Anesthesia and Intensive Care Medicine

Humanitas Clinical and Research Center - IRCCS

Via Manzoni 56, 20089 Rozzano (MI)

Italy

email: luca.carenzo@humanitas.it

APPENDIX A

Questionario di Feedback per il Medico ed il collega Non Medico

Nel rispondere a questo questionario apponi una croce sulla risposta che ritieni meglio rappresenti il collega che ti ha chiesto di compilarlo.

Il questionario è anonimo per chi compila

Nome del medico

Sono un collega (per favore apponi una croce) MEDICO / NON MEDICO

Per favore valuta da 1 a 5 le seguenti voci dove 1 rappresenta una costante performance sotto la media mentre 5 una costante performance sopra la media.

Buona Pratica Clinica

| E’ competente nel diagnosticare i problemi del paziente | | | | | |
| --- | --- | --- | --- | --- | --- |
| 1. Per Nulla | 2. | 3. | 4. | 5. Molto | Non So |
|  |  |  |  |  |  |
| E’ competente dal punto di vista tecnico (skill tecniche e manuali) – in relazione al livello di formazione | | | | | |
| 1. Per Nulla | 2. | 3. | 4. | 5. Molto | Non So |
|  |  |  |  |  |  |
| Sa giudicare rischi e benefici nel trattamento dei pazienti | | | | | |
| 1. Per Nulla | 2. | 3. | 4. | 5. Molto | Non So |
|  |  |  |  |  |  |
| Risponde appropriatamente al dolore e al distress dei pazienti | | | | | |
| 1. Per Nulla | 2. | 3. | 4. | 5. Molto | Non So |
|  |  |  |  |  |  |

Comunicazione. (Relazione con i pazienti, i parenti e i colleghi)

| E’ in grado di comunicare efficacemente con i pazienti | | | | | |
| --- | --- | --- | --- | --- | --- |
| 1. Per Nulla | 2. | 3. | 4. | 5. Molto | Non So |
|  |  |  |  |  |  |
| E’ in grado di comunicare efficacemente con i colleghi | | | | | |
| 1. Per Nulla | 2. | 3. | 4. | 5. Molto | Non So |
|  |  |  |  |  |  |
| E' di in grado di effettuare consegne chiare ed efficaci funzionali alla prosecuzione delle cure del paziente | | | | | |
| 1. Per Nulla | 2. | 3. | 4. | 5. Molto | Non So |
|  |  |  |  |  |  |
| E’ in grado di fornire suggerimenti e feedback (onesti e costruttivi) | | | | | |
| 1. Per Nulla | 2. | 3. | 4. | 5. Molto | Non So |
|  |  |  |  |  |  |

Professionalismo (Etica, Responsabilità, Onestà)

| Rispetta i pazienti e la loro privacy | | | | | |
| --- | --- | --- | --- | --- | --- |
| 1. Per Nulla | 2. | 3. | 4. | 5. Molto | Non So |
|  |  |  |  |  |  |
| Sa chiedere aiuto | | | | | |
| 1. Per Nulla | 2. | 3. | 4. | 5. Molto | Non So |
|  |  |  |  |  |  |
| Sa riconoscere i propri limiti | | | | | |
| 1. Per Nulla | 2. | 3. | 4. | 5. Molto | Non So |
|  |  |  |  |  |  |
| Sa assumersi le proprie responsabilità | | | | | |
| 1. Per Nulla | 2. | 3. | 4. | 5. Molto | Non So |
|  |  |  |  |  |  |
| E’ affidabile | | | | | |
| 1. Per Nulla | 2. | 3. | 4. | 5. Molto | Non So |
|  |  |  |  |  |  |

Management (Gestione del team, Leadership, Pianificazione, Economia)

| E’ in grado di lavorare efficacemente in un ambiente complesso | | | | | |
| --- | --- | --- | --- | --- | --- |
| 1. Per Nulla | 2. | 3. | 4. | 5. Molto | Non So |
|  |  |  |  |  |  |
| E’ in grado di gestire efficacemente il tempo e le priorità | | | | | |
| 1. Per Nulla | 2. | 3. | 4. | 5. Molto | Non So |
|  |  |  |  |  |  |
| Usa le risorse in maniera appropriata | | | | | |
| 1. Per Nulla | 2. | 3. | 4. | 5. Molto | Non So |
|  |  |  |  |  |  |
| E’ in grado di assumere un ruolo di leadership quando necessario | | | | | |
| Per Nulla | 2. | 3. | 4. | 5. Molto | Non So |
|  |  |  |  |  |  |

Commenti
