# Supplementary material for: Assessing anaesthesiology and intensive care specialty physicians: An Italian language multisource feedback system
Source: PLoS One. 2021 Apr 23;16(4):e0250404. doi: 10.1371/journal.pone.0250404 (PMC8064525; doi:10.1371/journal.pone.0250404)
Supplement: S2 File — (DOCX) [file pone.0250404.s002.docx]

**Assessing Anaesthesiology and Intensive Care Specialty Physicians: An Italian Language Multisource Feedback System**

Luca Carenzo^1^, Tiziana Cena^2^, Fabio Carfagna^3^, Valentina Rondi^4^, Pier Luigi Ingrassia^5,6^, Maurizio Cecconi^1,3^, Claudio Violato^7^, Francesco Della Corte^2,4^, Rosanna Vaschetto^2,4^,

1. Department of Anesthesia and Intensive Care Medicine, Humanitas Clinical and Research Center - IRCCS, Via Manzoni 56, 20089 Rozzano (MI), Italy
2. Department of Anaesthesia and Intensive Care Medicine, Azienda Ospedaliero-Universitaria “Maggiore della Carità”, Via Mazzini 18, 28100 Novara, Italy
3. Humanitas University, Department of Biomedical Sciences, Via Rita Levi Montalcini 4, 20090 Pieve Emanuele – Milan, Italy.
4. Dipartimento di Medicina Traslazionale, Università del Piemonte Orientale, Via Solaroli 17, 28100 Novara, Italy
5. Centro di Simulazione, Centro Professionale Sociosanitario, Via Ronchetto 14, 6900, Lugano, Switzerland
6. Centro Interdipartimentale di Didattica Innovativa e di Simulazione in Medicina e Professioni Sanitarie, SIMNOVA, Università del Piemonte Orientale, Via Lanino 1, 28100 Novara, Italy.
7. Departments of Medicine and Medical Education, University of Minnesota Medical School, 420 Delaware St. SE, Minneapolis, MN, USA, 55455

Corresponding author:

Luca Carenzo,

Department of Anesthesia and Intensive Care Medicine

Humanitas Clinical and Research Center - IRCCS

Via Manzoni 56, 20089 Rozzano (MI)

Italy

email: luca.carenzo@humanitas.it

APPENDIX B

Questionario di Feedback per il Paziente

Nel rispondere a questo questionario croci la risposta che meglio rappresenta la sua situazione o punto di vista. Le risposte che fornisce riguardano il suo incontro di oggi con il medico anestesista-rianimatore.

Per favore NON scriva il suo nome su questo foglio. Il questionario è anonimo e lei non sarà identificabile. Le informazioni saranno fornite al suo anestesista in forma aggregata.

Nome del Medico Dr.____________________________________________

Data_______/__________/__________

Compilo questo questionario per

A) Me Stesso

B) Mio Figlio

C) Un mio parente, compagno o partner

Nel caso abbia risposto B o C per favore compili questo questionario dal punto di vista del paziente.

2) Qual è il motivo per cui ha visto l’anestesista-rianimatore oggi?

A) Sono operato oggi o domani

B) Ho ricevuto analgesia per il mio parto

C) Sono ricoverato in Terapia Intensiva/Subintensiva

D) Ho fatto il pre-ricovero per un intervento chirurgico

E) Sono un paziente dell’ambulatorio di terapia antalgica

Come valuta l’anestesista-rianimatore che la ha vista oggi?

Risponda con una sola risposta da 1 a 5 oppure Non So

|  |  |  |  |  |  |  |
| --- | --- | --- | --- | --- | --- | --- |
|  | 1 Scadente | 2 | 3 | 4 | 5 Ottimo | Non So |
| a. Si è presentato |  |  |  |  |  |  |
| b. E’ stato cortese |  |  |  |  |  |  |
| c. Mi ha messo a mio agio |  |  |  |  |  |  |
| d. E’ stato scrupoloso nella visita |  |  |  |  |  |  |
| e. Le spiegazioni sono state chiare |  |  |  |  |  |  |
| f. Mi ha reso partecipe delle decisioni circa la mia anestesia/terapia |  |  |  |  |  |  |
| g. Ha risposto ai miei dubbi/domande |  |  |  |  |  |  |

4) Per favore valuti quanti è in accordo o in disaccordo con le seguenti affermazioni

|  |  |  |  |  |  |  |
| --- | --- | --- | --- | --- | --- | --- |
|  | 1  Per nulla d’accordo | 2 | 3 | 4 | 5  Molto in accordo | Non So |
| a. L’anestesista/rianimatore mi sembra disponibile |  |  |  |  |  |  |
| b. Ho l’impressione che l’anestesista/rianimatore sia in grado di fornire cure e procedure in sicurezza |  |  |  |  |  |  |
| c. Sono soddisfatto delle cure ricevuto dall’anesista/rianimatore e sarei felice di riaverlo come medico |  |  |  |  |  |  |
| d. L’anestesista/rianimatore mi ha trattato con dignità e rispetto |  |  |  |  |  |  |
| e. L’anestesista/rianimatore ha rispettato la mia privacy |  |  |  |  |  |  |
